# Rapid Sensing of Key Quality Components in Black Tea Fermentation Using Electrical Characteristics Coupled to Variables Selection Algorithms

**DOI:** 10.1038/s41598-020-58637-9

**Published:** 2020-01-31

**Authors:** Chunwang Dong, Ting An, Hongkai Zhu, Jinjin Wang, Bin Hu, Yongwen Jiang, Yanqin Yang, Jia Li

**Affiliations:** 1 Tea Research Institute, The Chinese Academy of Agricultural Sciences, Hangzhou, 310008 China; 2 College of Mechanical and Electrical Engineering, Shihezi University, Shihezi, 832003 China

**Keywords:** Electrophysiology, Computational models, Data processing, Machine learning, Quality control

## Abstract

Based on the electrical characteristic detection technology, the quantitative prediction models of sensory score and physical and chemical quality Index (theaflavins, thearubigins, and theabrownins) were established by using the fermented products of Congou black tea as the research object. The variation law of electrical parameters during the process of fermentation and the effects of different standardized pretreatment methods and variable optimization methods on the models were discussed. The results showed that the electrical parameters vary regularly with the test frequency and fermentation time, and the substances that hinder the charge transfer increase gradually during the fermentation process. The Zero-mean normalization (Zscore) preprocessing method had the best noise reduction effect, and the prediction set correlation coefficient (Rp) value of the original data could be increased from 0.172 to 0.842. The mixed variable optimization method (MCUVE-CARS) of Monte Carlo uninformed variable elimination (MC UVE) and competitive adaptive reweighted sampling (CARS) was proved that the characteristic electrical parameters were the loss factor (D) and reactance (X) of the low range. Based on the characteristic variables screened by MCUVE-CARS, the quantitative prediction models for each fermentation quality indicator were established. The Rp values of the sensory score, theaflavin, thearubigin and theabrownins of the predicted models were 0.924, 0.811, 0.85 and 0.938 respectively. The relative percent deviation (RPD) values of the sensory score, theaflavins, thearubigins and theabrownins of the predicted models were 2.593, 1.517, 1,851 and 2.920 respectively, and it showed that these models have good performance and could realize quantitative characterization of key fermentation quality indexes.

## Introduction

Congou black tea is a kind of unique tea in China^1^. Fermentation is the key process of black tea processing. Its essence is the process of forming the specific quality and flavor of the black tea generated by the polyphenolic compounds undergoing enzymatic oxidation to form theaflavins (TFs), thearubigins (TRs) and theabrownins (TBs) after the damage of the cell membrane of fresh tea leaves^2–4^. In the actual production of black tea, the grasp of the degree of fermentation quality is mainly controlled by the tea masters, which is susceptible to environmental, psychological and subjective experience^5^. Physical and chemical detection methods are required to analyze the changes of endogenous components in order to accurately grasp the fermentation quality, but physical and chemical detection has the disadvantages of long cycle, high cost and poor timeliness^6^.

Electrical measurement is a rapid non-destructive testing method that uses the electromagnetic characteristics of the substances that are to be tested to establish an intrinsic relationship with the contained components. K.H.Norian^7^ explained Tetraethylammonium(TEA) can selectively block K+ channels by establishing the charge control model of ion channels. Based on the permittivity and resistivity data of seven typical loess profiles, Zhang Song *et al*.^8^ summarized the dielectric and resistivity characteristics of loess and effectively distinguished the loess layer and red soil layer. The electrical measurement is also widely used in the detection of food quality or material property, and has the advantages of stability, rapidity, sensitivity, and low cost^9,10^. The electrical characteristic parameters mainly include capacitance, resistance, reactance, loss factors and impedance^11–14^. In fact, the cell structure and tissue structure of an organism are very complicated. They are mostly composed of dielectrics, conductors and electrolytes in various forms. When the organism is placed in an electric field, the bound charge in the material molecule will respond to the applied electric field. The contained components are equivalent to the dielectric, and also affect the electrical effects such as impedance and capacitive reactance^15^. At present, the electrical characteristic detection technology has been widely used in the quality composition and quality monitoring of agricultural products such as fruits, vegetables and cereals^16^. Chan, C.S. *et al*.^17^ used apparent soil electrical conductivity model to predict paddy productivity. Urska K. *et al*.^18^ established a linear regression model for the relationship between electrical conductivity and ash of Slovenian honey to determine the botanical origin of honey. The tea leaves are between conductors and insulators, and it would exhibit an electrical effect under an external electric field.

Japanese scholar Mizukami *et al*.^19^ established the characterization method of the decoction degree and water content of steamed green tea based on the detection technology of electrical impedance spectroscopy. In China, Yan Jianwei *et al*.^20^ studied the dielectric characteristics of the finished tea under different test conditions, and concluded the law of influence that temperature, frequency and water content have on the dielectric characteristics, and at the same time provided a theoretical basis for the non-destructive testing of the water content of finished tea. Feng Chengyan *et al*.^21^ analyzed the effects that tea variety, leaf position, water content and tenderness have on the dielectric characteristics of fresh leaves with the use of LCR digital bridges, indicating that the capacitances and dielectric constants of different tea varieties are significantly different from each other, that water content of fresh leaves is positively correlated with the capacitances and dielectric constants and that the capacitances and dielectric constants of the pathological fresh leaves and tea stems are significantly higher than those of normal tea leaves.

To sum up, there are few studies on the electrical characteristics detection technology in the field of tea, and especially the application of the testing of the fermented products of black tea has never been reported^22–25^. In order to overcome the deficiencies of artificial sensory evaluation and physical and chemical testing, and improve the timeliness, rapidity and accuracy of the existing methods for detecting the fermentation quality of black tea. In this thesis, the relationship between the six electrical parameters such as parallel equivalent capacitance (Cp), complex impedance (Z), resistance (R), reactance (Χ), loss factor (D) and phase loss angle (θ) of fermented samples and the sensory quality of the black tea and the main physical and chemical quality indicators were studied by taking the fermented products of Congou black tea as research object. Chemometrics is used to screen the sensitive electrical parameters and their excitation frequencies and establish a quantitative evaluation model of fermentation quality based on electrical characteristic parameters. This study provides new ideas and methods for rapid non-destructive testing of fermentation quality and provides a theoretical basis for further realization of intelligent and precise processing of fermentation quality.

## Materials and Methods

### Experimental material

The experiment was carried out in Sichuan Chuanhong Tea Group (Linhu Base) in September 2018. The Fuding white variety was selected as the raw material of fresh tea leaves. One bud and one leaf were picked as the tenderness, and the process conditions such as withering, twisting, fermentation and drying were consistent. The fermentation test was carried out with the use of a drum type black tea fermentation machine, the fermentation temperature was set to 30 degrees, and the relative humidity was set to ≥90%. The fermentation cycle of black tea is usually 3 to 4 hours. In order to improve the generalization of the predicted model, the fermentation cycle was extended to 6 hours so that the sample set can cover different fermentation stages (slightly fermented, moderate fermented and excessive fermented). During the process of fermentation, sampling 1 times per 30 min, 6 samples were taken at a time from different places, and a single sample was 200 g. A total of 78 samples were obtained during the process of fermentation for electrical characteristic detection, physical and chemical detection and sensory evaluation.

### Electrical characteristic information collection

**Figure 1:**
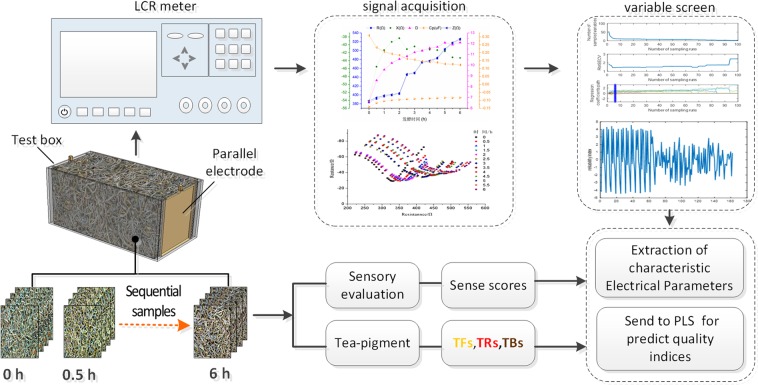
Electrical characteristics test platform and terminal interface.

The electrical characteristic detection system consists of 2816B type LCR bridge tester (manufactured by Changzhou Teng-hui Electronics Co., Ltd., China), test electrode, test box, host and acquisition software. The testable frequency range of the tester is 50 Hz to 200 kHz, and the test electrode is a self-made parallel plate electrode with the pitch of 10 cm. The material is gold-plated copper and the area of the electrode plate is 20 cm^2^ (length, width, thickness are 5 cm, 4 cm and 1 mm respectively). The LCR tester exchanges data with the host through the R232 serial port and connects the two test electrodes through the twisted pair shielded wire. Automatic detection, transmission and recording of electrical signal data are realized through the terminal acquisition software. The process of information collection and analysis is shown in Fig. 1.

Preheat the LCR tester for 30 minutes, and single-frequency open correction and single-frequency short-circuit correction were performed, 50 g of the sample to be tested was weighed and evenly loaded into the test box to test the electrical parameters of the sample. The test voltage of the electrical characteristic parameter was set to 1 V at 27 typical frequency points (0.05 kHz, 0.06 kHz, 0.08 kHz, 0.1 kHz, 0.2 kHz, 0.3 kHz, 0.4 kHz, 0.5 kHz, 0.6 kHz, 0.8 kHz, 1 kHz, 2 kHz, 3 kHz, 4 kHz, 5 kHz, 6 kHz, 8 kHz, 10 kHz, 20 kHz, 30 kHz, 40 kHz, 50 kHz, 60 kHz, 80 kHz, 100 kHz, 150 kHz and 200 kHz) to test the six electrical parameters, including parallel equivalent capacitance (Cp), complex impedance (Z), resistance (R), reactance (Χ), loss factor (D) and phase loss angle (θ), and each sample corresponds to 162 (27 × 6) electrical parameter values.

### Physical and chemical composition measurement

The samples were pretreated with lyophilization and milling, and the content of theaflavins were measured according to the national standard method *GB/T 30483-2013 Measurement of Theaflavins in Tea - High Performance Liquid Chromatography*. The determination of the content of thearubigins and theabrownins were carried out with reference to the system analysis method^26–28^.

### Sensory evaluation method

These samples during the process of fermentation are made into black tea after drying, and three national senior tea masters conduct a sensory evaluation of the tea samples based on GB/T 23776-2009. The specific operation is as follows: 3 g of dry tea was brewed with 150 ml pure water for 5 min, then observed the shapes of the dry tea leaves, evaluated the color, aroma, taste and leaf bottom of the tea, and calculated the total score of the evaluation. The average of the three total scores was calculated as the final sensory score.

### Data processing and analysis

After the completion of the electrical characteristic parameter detection, the electrical characteristic parameters were standardized pretreatment with 6 methods, including Multiplicative scatter correction (MSC), Savitzky-Golay (S/G), Min-Max normalization (Min-Max), Smooth and Zero-mean normalization (Zscore). The correction set and the prediction set samples were divided by the Kennard-Stone (K-S) method. Pick out 55 samples from the 78 samples collected with the K-S method as the correction set, and the remaining 23 samples were used as the prediction set. The ratio between the correction set and the prediction set was 7:3.

In order to extract the characteristic variables related to the target from the electrical parameter variables at multiple test frequencies, we use the method of characteristic wavelength optimization in near-infrared spectrum for reference, and compare the test frequencies with the electrical parameter variables to the wavelength and absorbance of the near-infrared spectrum, respectively. Monte Carlo Uninformed Variable Elimination Method and competitive adaptive weight sampling method are the two typical characteristic wavelength optimization algorithms in near-infrared spectroscopy. In this paper, the combination of two algorithms (MCUVE-CARS) is adopted to optimize the electrical characteristic parameters and eliminate useless characteristic variables^29^. A PLS predicted model of multiple quality indicators was established based on the preferred electrical parameters.

Generally, the models with higher accuracy and generalization have higher correction set correlation coefficient (Rc) and prediction set correlation coefficient (Rp), and smaller correction set cross-validation root mean square error (RMSECV), verification set prediction of root mean square error (RMSEP) and principal component number (PCs). Relative analysis deviation (RPD) is the ratio of standard deviation to RMSEP, which can be used as the final evaluation indicator to better measure the predictive ability of the established model^30^. When RPD is bigger than 2, it indicates that the model has excellent prediction ability^31^. When RPD is between 1.8 and 2.0, it indicates that the model is effective and can be used for quantitative analysis of the samples. When RPD is between 1.4 and 1.8, it indicates that the model can be used for rough prediction and correlation evaluation of the samples. When RPD is smaller than 1.4, it indicates that the model is very poor.

All data are processed on MATLAB® 2017b (Math Works, Natick, USA) and Microsoft Windows 7 (64-bit) platforms.

## Results and Discussion

### Performance test of parallel electrode

At different fermentation time points, the electrical parameters of the same sample at different test frequencies were repeatedly collected. Each sample was repeatedly tested 6 times, and the relative standard deviation (RSD) values of the 6 sets of data were calculated. As shown in Table S1, all RSD values are less than 1%, and the interval is 0.001–0.0513.The test accuracy of the high frequency region is better than the low frequency region. In conclusion, the electrode detection system of this experiment has good repeatability and stability.

### Change of electrical parameters at different test frequencies

**Figure 2:**
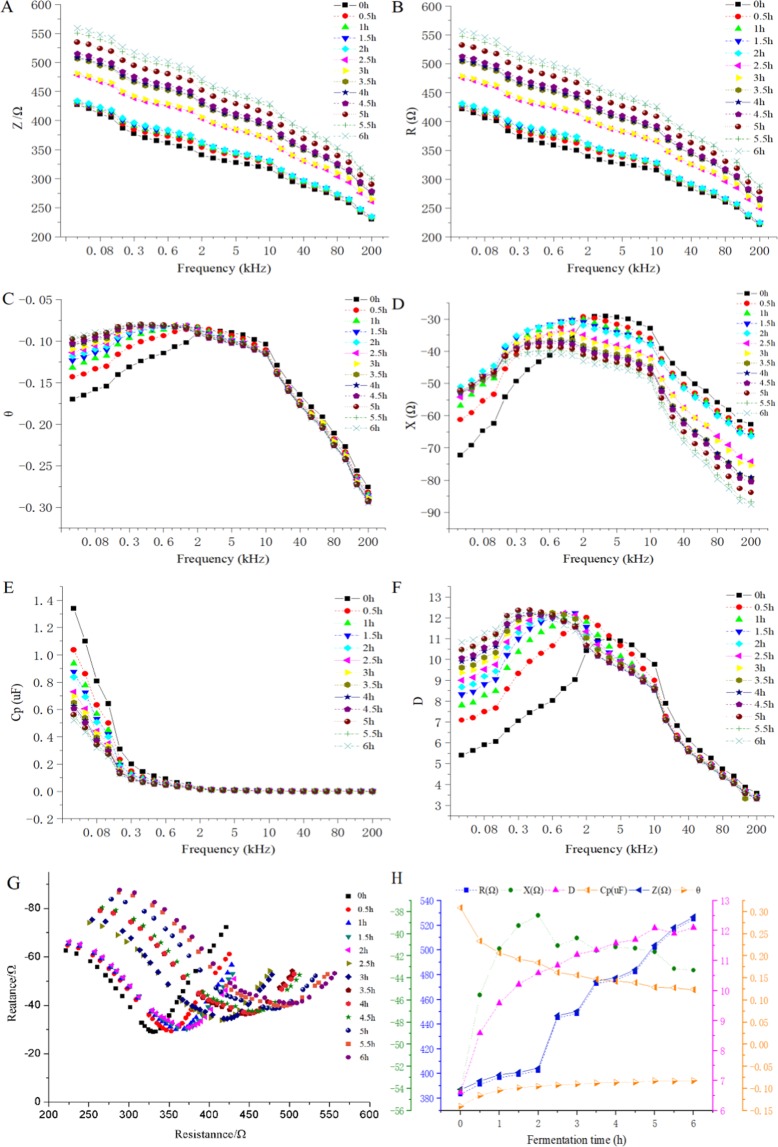
Effect of test frequency on electrical parameters of black tea fermentation samples.

Figure 2 shows the effect of the test frequency on the electrical parameters of the fermented samples of the black tea. It can be seen from Fig. 2A,B that the value Z and value R of the fermented samples of the black tea gradually decrease with the increase of the frequency, and the fermentation time has a great influence on the frequency characteristics of the electrical parameters, both of which gradually increase with the process the fermentation.

Figure 2C shows that the value θ increases first and then decreases with the increase of the frequency, and it varies greatly in the low frequency range. After 2 kHz, the magnitude of the change in the value θ narrows sharply. As shown in Fig. 2F, the value X increases first and then decreases with the increase of the frequency, and it varies greatly in the middle and low frequency range. The fermentation time has a great influence on the frequency characteristics of value X. In the low frequency range, it gradually increases with the process of fermentation, and gradually decreases with the fermentation time in the high frequency range (as shown in Fig. 2D).

Figure 2E shows that the value Cp decreases sharply with the increase of frequency, and then the magnitude of the change narrows sharply after 300 Hz, and tends to be stable afterwards. The fermentation time has a great influence on the value Cp in the low frequency range and varies greatly in the low frequency range. It can be seen from Fig. 2F that the value D increases first and then decreases with the increase of frequency, varies greatly in the low frequency range, and the magnitude of the change narrows sharply after 10 kHz.

The effect curve of the test frequency on the resistance-reactance are shown as Fig. 2G. It can be seen from Fig. 2B,D that the value X of the fermented samples of the black tea first increases and then decreases with the increase of the value R, and the different fermentation times have the similar change trend. As the resistance-reactance amplitude of the process of fermentation (the minimum absolute value of X in the spectrum) gradually increases, the amplitude increases from 29.06 Ω to 34.43 Ω when the fermentation time is at the 3^rd^ hour (2.5^th^ hour and 3^rd^ hour almost overlap), an increase of 11.35% compared to that at the 2^nd^ hour.

To sum up, the detection frequency has a significant effect on the electrical parameters of the fermented samples of the black tea, and the fermentation time has a greater impact on characteristics of the electrical parameters in the low frequency range, which is because the electrical characteristics of substances are closely related to frequency and its structure^32^. The cells are closer to the insulators at low frequencies, so the impedance phase (Z, R, X) is relatively high and θ is relatively small when the current flows around the cell membrane, at the same time, the charge accumulates at the edge of the conductive region and the conductivity of the ions makes the capacitance higher. As the frequency increases, the capacitive reactance of the cell membrane gradually appears, both of value θ and value D increases. The impedance is relatively small when the current flow through the cell membrane at high frequencies, which causes the value of θ and D decreases gradually with the decrease of cell membrane reactance. At this time, the orientation polarization of the dipole gradually stops, showing a decrease in Cp.

In addition, the resistance-reactance curve can be fitted to an arc with the opening facing down when the organism has a complete cell structure^33^. During the process of fermentation of the black tea, along with the formation of some macromolecular compounds (such as TRs and TBs), the electrical parameters vary with the fermentation time^28^.

### Law of change of electrical parameters in different fermentation stages

Taking the characteristic frequency of 200 kHz as an example, the law of change of each electrical characteristic parameter of the black tea in different fermentation stages is shown in Fig. 2H. With the extension of fermentation cycle, the impedance, reactance, impedance angle and loss factor gradually increase. The reactance reaches the peak after 2 hours of fermentation, and then gradually decreases. The impedance angle tends to be stable after 2 hours of fermentation. The capacitance gradually decreases with the extension of fermentation cycle. As the capacitance decreases, the impedance and loss factor increase, this indicates that there are more substances that hinder the transfer of electric charge during the process of fermentation.

In addition to the significant influence of frequency, the dielectric characteristics of the substances are also closely related to its contained composition and structure^32^. The effect that fermentation time on electrical parameters may be related to the physiological and biochemical changes of the samples during the process of fermentation. The fermented samples of the black tea can be regarded as a bioelectric field with charged particles.

The vibrating section method is used to collect the cell sections of the twisted leaves and fermented leaves. The 40-fold microstructure of the light microscope of the sample cell section is shown in Fig. S1. After the tea leaves are twisted, the palisade tissue and the sponge tissue cells are damaged, and the pectin (tea juice) in the cytochylema is released. At the same time, with the oxidative polymerization of polyphenols in the tea juice during fermentation, polymer compounds such as TFs, TRs, and TBs are formed^19^ so that the cell tissues in the section appear “chartreuse”. The above changes in cell structure and composition cause an increase in impedance, a decrease in capacitance, and a decrease in dielectric loss factor in fermentation^34^.

### Optimization of the pretreating method of electrical parameter data

Because of the different dimensions, magnitudes and sensitivities of the electrical parameters at each test frequency, the standardization method is adopted to pretreat the raw data of various types of sensors so that various kinds of electrical parameter data can be converted to the same order of magnitude and the influence of the dimensions can be removed. The electrical parameter data were processed by MSC, Smooth, Min-Max and Zscore, etc., and then the PLS model was established based on the processed data. The performance of each model is shown in Table S1. The results show that Zscore is the best pretreating method for electrical characteristic parameters, which improves the Rp value from 0.172 to 0.842 of the original data, and is significantly higher than other pretreating methods. RMSEP is 2.194 and system deviation (Bias) is 0.232, which is also significantly lower than other pretreating methods. In addition, the difference between RMSECV and RMSEP of the Zscore model is 1.157, which is the smallest among all pretreating methods, indicating that the Zscore standardization process makes the model have better generalization performance.

### The optimization of characteristic electric parameter and prediction model

#### MCUVE-based characteristic electric parameter optimization and prediction model

The Monte Carlo Uninformed Variable Elimination Method (MCUVE) is an improved method based on the Uninformed Variable Elimination Method (UVE), which evaluates the importance of each variable in the model by calculating the Stability Index (RI) of each variable^35^. The values of RI are used to sequence the variables, and a new set of variables is established accordingly. Then, one variable is gradually added to establish the PLS model, and the minimum RMSEP value of the prediction set is used as the evaluation index of the variable retention number^36^.

**Figure 3:**
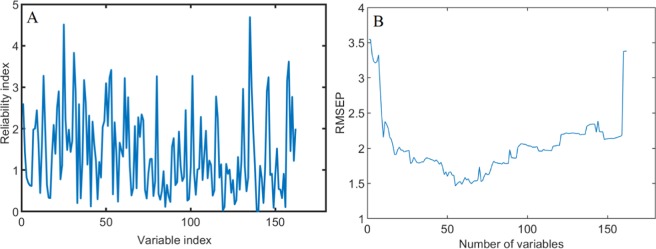
Optimizing the characteristic electrical parameters of sensory score using MCUVE.

Taking the sensory quality-related characteristic variable screening as an example, as shown in Fig. 3A, the RI values of all the electric parameters in the range of 162 electric characteristic variables are given. The larger the amplitude, the better the point. As can be seen from Fig. 3B, the minimum value of RMSEP is 1.463 for the model of sensory evaluation, and the corresponding abscissa point is 54, that is to say, the number of reserved variables is 54. When the number of reserved variables is bigger than 54, the RMSEP value increases rapidly with the increase of the number of reserved variables, which means that when fewer variables are retained, the useful variables information variables cannot be all included in the model, and when more reserved variables are retained, the useless variable information will also affects the prediction results.

The selection results of MCUVE variables are summarized (as shown in Table S2). The results show that the frequency of the selected stability index (RI) of sensory score bigger than 4 is mainly concentrated in the low frequency range (0.06–0.4 kHz), and the variables with high stability are the loss factor (D) and reactance (X) of the electrical parameter, where the RI value of the variable D is the highest (4.53) at the frequency of 0.4 kHz. In terms of the optimization of electrical parameters related to physical and chemical components, the frequency of the selected stability index (RI) by TFs bigger than 2 is mainly concentrated in the middle and low frequency ranges (0.1–5 kHz). The variables with high stability are the loss factor (D) and reactance (X) of electrical parameters, where the RI value of the variable X is the highest (3.17) at the frequency of 1 kHz. The frequency of the selected stability index (RI) by TRs bigger than 2.5 is mainly concentrated in the middle and low frequency ranges (0.1–5 kHz). The variables with high stability are the loss factor (D) and reactance (X) of the electrical parameters, where the RI value of the variable X is the highest (3.47) at the frequency of 0.6 kHz. The frequency of the selected stability index (RI) by TBs bigger than 3.7 is also mainly concentrated in the low frequency range (0.05–0.1 kHz). The variables with high stability are the loss factor (D) and reactance (X) of the electrical parameters, where the RI value of the variable X is highest (4.51) at the frequency of 0.1 kHz. To sum up, as is analyzed above, the stable test frequency with each quality index is the low frequency range, and the most relevant characteristic electrical parameters are D and X.

#### MCUVE-CARS-based characteristic electrical parameter optimization and prediction model

Competitive adaptive reweighted sampling (CARS) is a new variable selection method proposed in recent years, which can simplify and improve the prediction accuracy of the models and is suitable for variable selection of high-dimensional data^37^. The algorithm eliminates a great amount of irrelevant information, but the number of variables is still very large. The CARS is adopted to further optimize the electrical parameters selected by MCUVE to obtain the collinear minimum effective wavelength so as to establish a more compact and stable PLS correction model.

**Figure 4:**
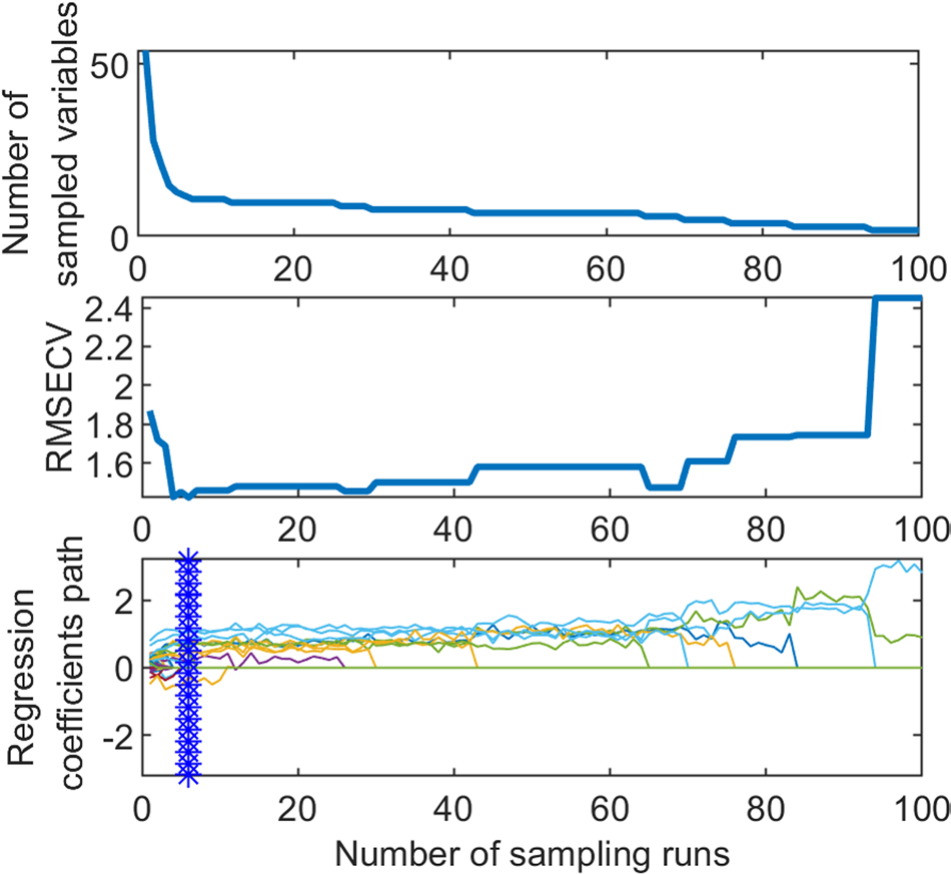
Optimizing the characteristic electrical parameters of sensory score using MCUVE-CARS.

In order to eliminate the adaptive weighted sampling error of the CARS algorithm, the number of continuous samples of the CARS algorithm is set to 50 and 10 for the interactive verification, and the corresponding variables subset to the minimum RMSECV is selected as the characteristic variable. Figure 4 shows the selection process of the MCUVE-CARS characteristic electrical parameter of the sensory score prediction model (where ‘*’ represents the position of the minimum RMSECV value and the number of screening variables). The results show that when the number of samples is 8, the RMSECV reaches the minimum value (1.235), and the corresponding characteristic electrical parameters are 10. The characteristic variables of each quality index screened by the MCUVE-CARS method are shown in Table S3. Taking the sensory score as an example, the PLS prediction model of sensory score was established with the use of 10 characteristic electrical parameters selected by MCUVE-CARS. When the number of the principal component is 3, Rp and RESEP are 0.924 and 1.325 respectively, and the RPD is 2.593 (bigger than 2.5), indicating that the model has very good predictive ability and can be used for quantitative analysis and quality control in the practice of production.

**Figure 5:**
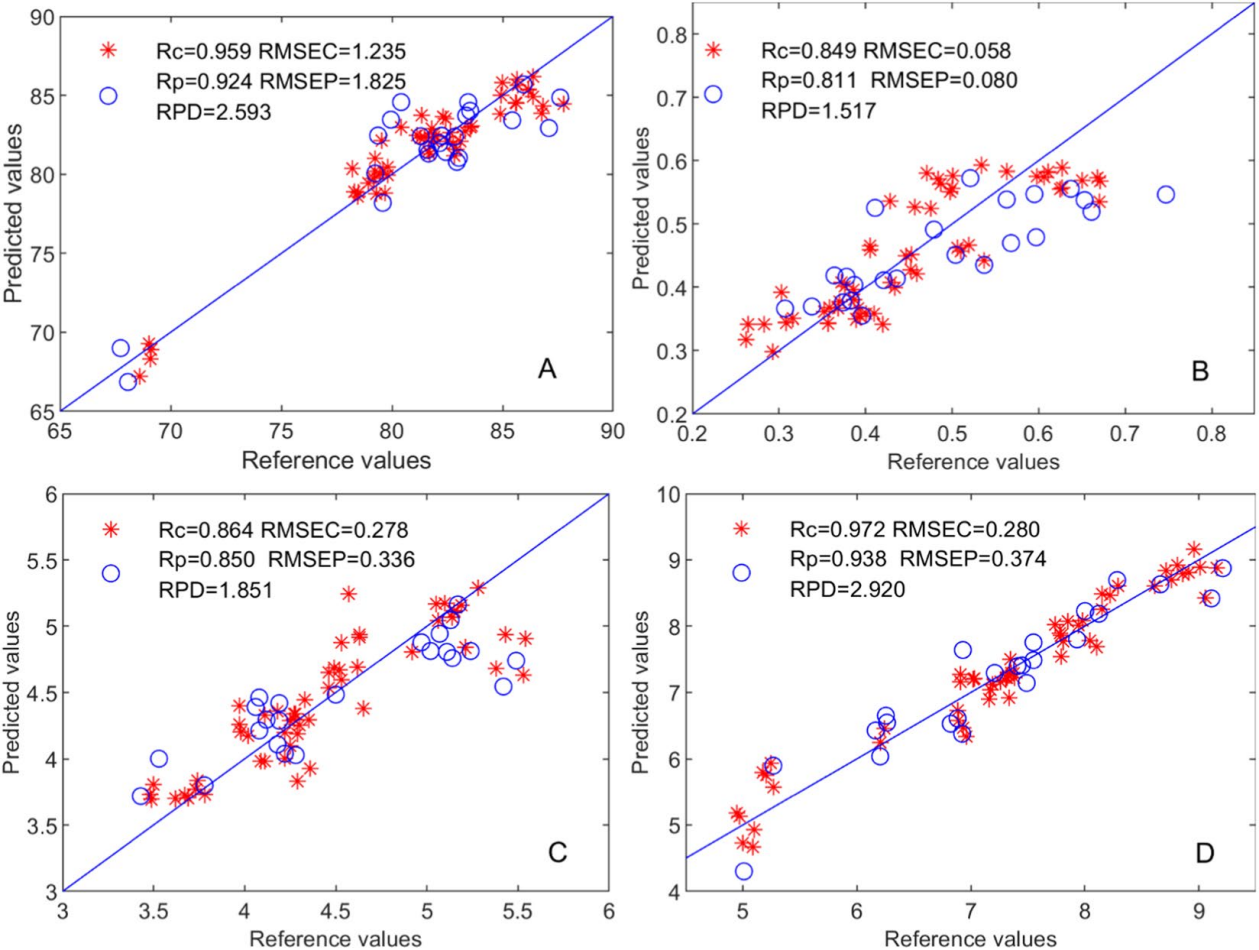
Scatter of predicted values and experimental values in models for quality indicators.

The scatter figure of the predicted values and experimental values of the MCUVE-CARS-PLS models of sensory score, TFs, TRs, and TBs are shown in Fig. 5A–C, and Fig. 5D respectively. Table 1 shows the analysis results of various fermentation quality indicators of the black tea established by different PLS models. The combined results show that the MCUVE-CARS-PLS prediction models of all the quality indicators all have good performance for quantitative analysis.

**Table 1:** Results from different models for predicting quality indicators in black tea.

Quality index	Method	Variable number	PCs	Calibration set		Prediction set				
				*Rc*	*RMSECV*	*Rp*	*RESEP*	*bias*	*RSD*	*RPD*
Sensory score	PLS	162	3	0.974	1.037	0.842	2.194	−0.32	4.160	1.556
	MCUVE-PLS	54	3	0.96	1.209	0.901	1.463	0.104	5.578	2.182
	CARS-PLS	28	3	0.971	1.091	0.898	1.798	−0.201	5.948	1.804
	**MCUVE-CARS-PLS**	**10**	**3**	**0.959**	**1.235**	**0.924**	**1.335**	**0.077**	**5.819**	**2.593**
Theaflavins	PLS	162	6	0.838	0.06	0.794	0.081	0.029	13.616	0.893
	MCUVE-PLS	34	2	0.897	0.059	0.731	0.091	0.042	10.713	1.199
	CARS-PLS	24	7	0.882	0.021	0.709	0.092	0.033	10.687	1.126
	**MCUVE-CARS-PLS**	**8**	**1**	**0.849**	**0.058**	**0.811**	**0.080**	**0.031**	**9.561**	**1.517**
Thearubigins	PLS	162	2	0.796	0.334	0.813	0.362	0	10.385	1.177
	MCUVE-PLS	16	3	0.846	0.309	0.829	0.305	0.062	9.010	1.663
	CARS-PLS	17	3	0.88	0.262	0.742	0.409	0.011	9.946	1.214
	**MCUVE-CARS-PLS**	**5**	**3**	**0.864**	**0.278**	**0.85**	**0.336**	**0.071**	**8.912**	**1.851**
Theabrownins	PLS	162	5	0.896	0.393	0.918	0.536	0.017	12.354	1.552
	MCUVE-PLS	21	2	0.952	0.391	0.878	0.432	−0.011	12.727	2.137
	CARS-PLS	21	5	0.99	0.172	0.901	0.513	−0.169	10.607	2.204
	**MCUVE-CARS-PLS**	**6**	**2**	**0.972**	**0.28**	**0.938**	**0.374**	**0.016**	**9.966**	**2.920**

In addition, Dong Chunwang *et al*.^6,38^ carried out evaluation of fermentation quality indicators based on near-infrared and machine vision technology. Table S5 shows the results of comparison with the prediction results of electrical characteristics models. The results showed that the RPD values of the three models were comparable, and all of them could be used to detect the fermentation quality of black tea. The near-infrared spectroscopy method was superior.

## Conclusion

1. The law of change of the electrical characteristic parameters in the process of fermentation at each test frequency is clarified. During the process, the electrical impedance, reactance, impedance angle and loss factor all increase gradually, and the capacitance decreases with the extension of the fermentation cycle, indicating that more and more substances hinder the charge transfer during the process of fermentation.
2. Based on the MCUVE-CARS variable optimization method, the number of the selected characteristic electrical parameters of sensory score, TFs, TRs, and TBs are 10, 8, 5, and 6 respectively, the stable test frequency of each quality indicator is in the low frequency range, and the most relevant electrical parameters are D and X. The comparison of the model results shows that the variable screening method can effectively compress the variables and further improve the accuracy of the models.
3. A quantitative analysis model of PLS for predicting each fermentation quality indicator is established by the characteristic variables selected by MCUVE-CARS method. The RPD values of the predicted models of sensory score, TFs, TRs, and TBs are 2.593, 1.517, 1.851 and 2.920 respectively, indicating that the predicted models of all quality indicators all have good performance. The electrical characteristic detection technology can realize the rapid evaluation of the fermentation quality of Congou black tea.

## Supplementary information

Supplementary Information.
