# Supplementary material for: Rapid Sensing of Key Quality Components in Black Tea Fermentation Using Electrical Characteristics Coupled to Variables Selection Algorithms
Source: Sci Rep. 2020 Jan 31;10:1598. doi: 10.1038/s41598-020-58637-9 (PMC6994467; doi:10.1038/s41598-020-58637-9)
Supplement: Supplementary file 1 — Supplementary Information. [file 41598_2020_58637_MOESM1_ESM.pdf]

Supplementary Information

Title

Rapid Sensing of Key Quality Components in Black Tea Fermentation Using Electrical Characteristics Coupled to Variables Selection Algorithms

Author list

Chunwang Dong[a#], Ting An[a,b#], Hongkai Zhu[a], Jinjin Wang[a], Bin Hu[b], Yongwen Jiang[a], Yanqin Yang[a] and Jia Li [a*]*

Supplementary Figure

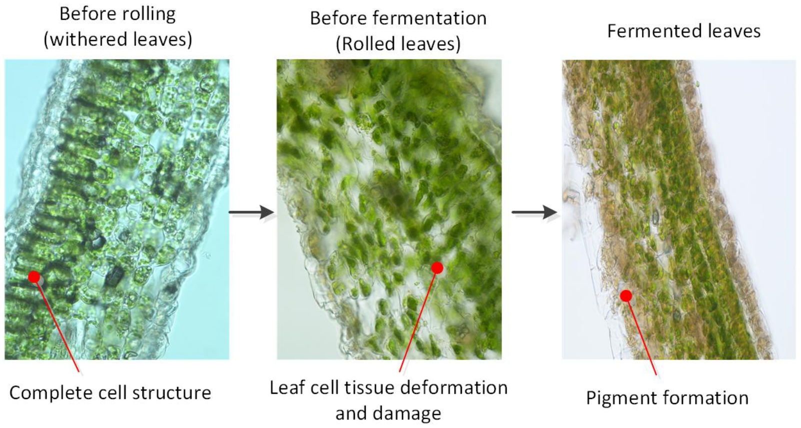

Fig. S1 Cell sections of leaves in wilting, rolling and fermentation

Supplementary Tables

Table S1 RSD (%) of parameter D in different fermentation time and frequency

Frequency (kHz)	Fermentation time（h）												
	0	0.5	1	1.5	2	2.5	3	3.5	4	4.5	5	5.5	6
0.05	0.085	0.015	0.005	0.009	0.004	0.007	0.006	0.004	0.006	0.002	0.010	0.003	0.007
0.06	0.037	0.126	0.166	0.151	0.150	0.094	0.140	0.139	0.112	0.106	0.114	0.125	0.108
0.08	0.092	0.141	0.163	0.149	0.174	0.100	0.179	0.181	0.130	0.168	0.164	0.124	0.189
0.1	0.141	0.190	0.164	0.177	0.188	0.141	0.175	0.188	0.130	0.143	0.163	0.148	0.128
0.2	0.486	0.513	0.388	0.426	0.348	0.331	0.304	0.320	0.217	0.289	0.312	0.261	0.170
0.3	0.218	0.256	0.181	0.256	0.182	0.196	0.130	0.159	0.106	0.119	0.122	0.137	0.099
0.4	0.167	0.138	0.098	0.142	0.088	0.119	0.102	0.085	0.057	0.085	0.064	0.043	0.032
0.5	0.126	0.145	0.073	0.096	0.064	0.065	0.055	0.047	0.038	0.044	0.037	0.025	0.027
0.6	0.095	0.065	0.050	0.075	0.044	0.049	0.031	0.036	0.019	0.022	0.023	0.018	0.011
0.8	0.099	0.072	0.040	0.037	0.031	0.039	0.026	0.013	0.003	0.013	0.013	0.011	0.010
1	0.058	0.053	0.026	0.033	0.020	0.018	0.007	0.008	0.006	0.007	0.004	0.008	0.009
2	0.129	0.033	0.017	0.030	0.010	0.018	0.006	0.007	0.007	0.006	0.012	0.006	0.016

3	0.042	0.012	0.006	0.005	0.006	0.007	0.005	0.015	0.009	0.011	0.009	0.008	0.012
4	0.010	0.007	0.008	0.007	0.011	0.012	0.018	0.012	0.015	0.012	0.013	0.015	0.019
5	0.011	0.007	0.013	0.007	0.008	0.007	0.011	0.016	0.017	0.016	0.011	0.010	0.019
6	0.004	0.009	0.013	0.009	0.012	0.008	0.008	0.012	0.011	0.017	0.011	0.006	0.017
8	0.247	0.011	0.009	0.008	0.008	0.006	0.008	0.010	0.009	0.007	0.008	0.011	0.013
10	0.369	0.007	0.005	0.010	0.006	0.008	0.013	0.008	0.008	0.007	0.007	0.007	0.009
20	0.460	0.004	0.003	0.006	0.008	0.007	0.007	0.006	0.004	0.009	0.008	0.002	0.012
30	0.493	0.003	0.004	0.006	0.004	0.004	0.005	0.008	0.004	0.004	0.005	0.005	0.006
40	0.011	0.003	0.003	0.006	0.002	0.004	0.004	0.003	0.003	0.003	0.002	0.005	0.003
50	0.007	0.004	0.005	0.009	0.003	0.003	0.003	0.001	0.004	0.004	0.002	0.003	0.005
60	0.010	0.004	0.007	0.003	0.003	0.008	0.002	0.001	0.004	0.003	0.001	0.003	0.005
80	0.007	0.005	0.005	0.004	0.002	0.010	0.003	0.002	0.002	0.003	0.002	0.003	0.002
100	0.009	0.005	0.003	0.002	0.004	0.005	0.002	0.003	0.002	0.002	0.002	0.003	0.001
150	0.005	0.002	0.001	0.003	0.004	0.004	0.006	0.004	0.005	0.004	0.003	0.002	0.003
200	0.007	0.003	0.002	0.004	0.003	0.004	0.048	0.004	0.002	0.003	0.002	0.002	0.002

Table S2 Effect of different pretreating methods on PLS regression of the models

Preparation method	PCS	Calibration set		Prediction set		
		Rc	*RMSECV*	*Rp*	*RMSEP*	*Bias*
Original data	2	0.523	3.571	0.172	4.898	0.548
MSC	2	0.523	4.100	0.020	3.579	0.393
Smooth	2	0.524	3.824	0.262	4.337	1.170
S/G 2st	2	0.515	3.856	0.244	4.238	0.881
**Zscore**	**3**	**0.904**	**1.037**	**0.842**	**2.194**	**0.232**
Min-Max	4	0.991	0.629	0.829	2.287	0.762

Table S3 Optimization of characteristic electric parameters by MCUVE

Quality index	Parameter	Characteristic variable*									
Sensory score	Frequency（kHz）	0.4	0.08	0.06	0.2	0.3	0.6	0.1	0.08	0.05	0.06
	Electrical parameters	D	D	D	X	D	D	X	X	X	X
	RI	4.53	4.39	4.28	4.17	4.16	4.07	4.04	4.04	4.03	3.98
TFs	Frequency（kHz）	1	2	0.1	1	30	0.6	0.5	0.3	0.4	5
	Electrical parameters	X	D	X	D	R	D	X	X	X	R
	RI	3.17	2.49	2.43	2.43	2.28	2.25	2.24	2.23	2.19	2.08
TRs	Frequency（kHz）	0.6	2	0.5	1	5	0.8	2	1	0.4	0.8
	Electrical parameters	D	D	X	D	R	X	X	X	X	D
	RI	3.47	3.32	3.06	2.96	2.92	2.78	2.74	2.51	2.51	2.45
TBs	Frequency（kHz）	0.1	0.3	0.08	0.06	0.05	0.08	0.5	0.5	0.1	0.06
	Electrical parameters	D	D	D	D	X	X	X	D	X	X
	RI	4.51	4.42	4.25	4.08	4.01	3.99	3.9	3.88	3.75	3.73

* Characteristic variables in the top 10;

Table S4 Results of characteristic electric parameters by MCUVE-CARS algorithm

Quality index	parameter	characteristic variable									
Sensory score	Frequency（kHz）	0.4	0.06	0.3	0.6	0.2	0.8	0.1	60	2	2
	Electrical parameters	D	D	D	D	D	D	D	θ	D	X
TFs	Frequency（kHz）	1	2	30	150	0.1	8	40	20		
	Electrical parameters	X	D	X	X	D	Cp	Cp	Z		
TRs	Frequency（kHz）	0.6	2	1	80	150					
	Electrical parameters	X	X	X	R	X					
TBs	Frequency（kHz）	0.3	0.1	0.08	0.06	0.5	0.6				
	Electrical parameters	D	D	D	D	X	X				

Table S5 Comparison of different detection methods for fermentation quality

Quality index	Method	Algorithm	Calibration set		Prediction set		
			Rc	*RMSECV*	*Rp*	*RESEP*	*RPD*
Sensory score	Electrical parameters	MCUVE-CARS-PLS	0.959	1.235	0.924	1.335	2.593
	Near infrared spectroscopy	SPA-GA-SVR	0.973	0.553	0.967	0.484	3.923
	Machine vision	RF	0.987	0.867	0.948	1.733	2.931
Theaflavins	Electrical parameters	MCUVE-CARS-PLS	0.849	0.058	0.811	0.08	1.517
	Near infrared spectroscopy	SPA-GA-SVR	0.861	0.039	0.833	0.047	1.77
	Machine vision	RF	0.97	0.033	0.891	0.058	1.612
Thearubigins	Electrical parameters	MCUVE-CARS-PLS	0.864	0.278	0.85	0.336	1.851
	Near infrared spectroscopy	SPA-GA-SVR	0.954	0.203	0.895	0.336	2.223
	Machine vision	RF	0.969	0.163	0.89	0.297	1.267
Theabrownins	Electrical parameters	MCUVE-CARS-PLS	0.972	0.28	0.938	0.374	2.92
	Near infrared spectroscopy	SPA-GA-SVR	0.987	0.306	0.984	0.278	5.462
	Machine vision	RF	0.986	0.168	0.944	0.347	2.636
